# Current Perspective of Plant-Based Diets on Communicable Diseases Caused by Viruses: A Mini Review

**DOI:** 10.3389/fnut.2022.786972

**Published:** 2022-03-16

**Authors:** Carisa Su-Ann Wong, Cheng Wei Lim, Haruna Isa Mohammed, Kong Yen Liew, Chau Ling Tham, Ji Wei Tan, Hui Yee Chee

**Affiliations:** ^1^School of Science, Monash University Malaysia, Selangor, Malaysia; ^2^Department of Biomedical Science, Faculty of Medicine and Health Sciences, Universiti Putra Malaysia, Selangor, Malaysia; ^3^Department of Microbiology, Nasarawa State University, Keffi, Nigeria; ^4^Department of Medical Microbiology, Faculty of Medicine and Health Sciences, Universiti Putra Malaysia, Seri Kembangan, Malaysia

**Keywords:** plant-based diet, SARS-CoV-2, hepatitis viruses, human papillomavirus, cancer

## Abstract

Communicable diseases are illnesses caused by pathogenic biological agents, including viruses, bacteria, fungi, parasites, and protozoa. Such diseases spread among people through contact with contaminated surfaces, bodily fluids, or blood products, or through the air, insect bites, or consuming contaminated food and beverages. Although some communicable diseases can be treated or prevented by taking medication and vaccines, there has been an increase in awareness of adopting a healthy diet to aid in the prevention and reversal of these diseases. One popular diet is a plant-based diet. Plant-based diets generally consist of vegetables, grains, nuts, seeds, legumes, and fruits, without any animal-source foods or artificial ingredients. Over the years, this diet has continuously increased in popularity. Reasons for following a plant-based diet are varied but include health benefits, such as improving immunity, and reducing the risk of heart disease, diabetes, and some cancers. Scientific evidence even shows that just an increased vegetable intake can decrease the occurrence of chronic diseases caused by viruses, such as hepatitis viruses, and reduce the risk of severe coronavirus disease 2019. Therefore, this mini review discusses the effectiveness of adopting a plant-based diet in ameliorating diseases caused by selected viruses and its limitations.

## Introduction

Infectious diseases account for approximately 20% of deaths in the world and viruses are responsible for at least one-third of these deaths (1). In the last decades, emerging and re-emerging viruses, such as the severe acute respiratory syndrome coronavirus (SARS-CoV), Ebola virus, Lassa virus, hepatitis viruses, human papillomavirus (HPV), and the most recent SARS-CoV-2, the virus causing coronavirus disease 2019 (COVID-19), have posed significant global public threats (2).

Several factors, including increasing population, poverty, and malnutrition, economic factors leading to population migration, unplanned urbanization, deforestation, increased domestic and global connectivity, social practices, prevalence of immunosuppressive diseases, and gene mutations in genetic sequences of pathogens, have been responsible for the increased susceptibility of humans to viral infections (3). The global negative impacts of these viruses, especially those of the current COVID-19, are tragic; besides the catastrophic death toll and large numbers of hospitalized patients pushing healthcare systems to the limit, the world is facing the immediate and large-scale shutdown of public life, services, production, trade, and travel, along with serious long-term effects, such as massive job losses and widespread recessions, all of which will have a profound impact for many years to come (4).

No doubt, vaccines have clearly been the game-changers in the fight against viral diseases. However, in the case of infections, such as those caused by the human immunodeficiency virus and hepatitis C virus (HCV), where vaccination has failed so far, alternative antivirals play a critical role in decreasing the morbidity and mortality related to those viral infections. Additionally, in most cases, the acceptance of vaccines for prophylaxis against these infections, particularly in developing countries, is not absolute (5). For instance, the approved Pfizer and Moderna COVID-19 vaccines by the Food and Drug Administration (FDA) have had several concerns including their safety, efficacy, and immunity development after administration. Hence, many people around the world have developed the habit of consuming plant-based diets as a trial for the purpose of maintaining their health and improving their immune system (6).

A plant-based diet is a diet consisting mostly or entirely of plant-based foods (7) and includes vegetables, grains, nuts, seeds, legumes, and fruits, with no animal-source foods or artificial ingredients. Although a plant-based diet is usually free of or has limited animal products, it is not necessarily vegan (8). From time immemorial, it is generally believed that plant-based diets are a source of essential nutrients that cannot be obtained from other food sources. The vitamins, macro-, micro-, and phytonutrients in plant-based diets, such as fruits, nuts, legumes, and vegetables, have been proven to improve the quality of health and immune responses. The micro- and phytonutrients, such as beta-carotene, vitamin C, vitamin E, and polyphenolic compounds, are those that confer antioxidant and anti-inflammatory properties, resulting in the modulation of our immune functions (9). Although previous studies have documented positive associations between the consumption of plant-based diet and outcomes of viral diseases and other health-related conditions, such as cancers, some studies still report otherwise. Hence, this mini review focuses on the reported outcomes of adopting a plant-based diet as well as a diet high in vegetables and fruits against some of the selected diseases caused by viruses, specifically SARS-CoV-2, hepatitis viruses, and HCV.

## SARS-CoV-2

Respiratory infectious diseases, including COVID-19, are the major infectious diseases that cause enormous mortality and morbidity globally (2). A worldwide shortage of vaccine supply, worry of the long-term side effects of COVID-19 vaccines, and limited FDA-approved oral antiviral drugs, such as Paxlovid and Molnupiravir (10), are among the factors causing people to practice non-pharmaceutical alternatives to improve their immunity and to prevent infection or becoming ill. One of the alternatives is to adopt a plant-based diet.

An extensive study by Yedjou et al. (6) demonstrated that a high intake of vegetables and/or fruits (VF) combats SARS-Cov-2 by preventing the incidence of COVID-19 and lowering the mortality rate (Table 1). The authors performed their study using a machine learning model to perform statistical analysis on the effects of VF consumption on COVID-19 incidences and deaths, utilizing diet data from the public database Kaggle, which involves 170 different countries. Their study revealed three major findings. First, in developed countries, people who ate lower quantities of VF had higher COVID-19 incidences and deaths. Second, in developed countries, where people consumed enough or more VF, the COVID-19 death rate was low even when the number of reported positive cases were high. Third, in developing countries, where people ate enough or more VF, the incidence and death rates of COVID-19 were lower. Another key finding was that when using a correlation-based feature selection (CFS) to study the correlation between VF intake and COVID-19 incidences or deaths, a negative association was observed. Interestingly, CFS was also used to identify the top nutritional factors that contribute to COVID-19 incidences and deaths, and their findings revealed that most factors were from animal products. A limitation of this study, however, is that as it was conducted using machine learning analysis, the study may be subjected to biases that cannot be accounted for because of the different geographical locations within a country that vary in VF intake or COVID-19 disease variation. Hence, further research is required to validate these findings.

**Table 1:** Clinical studies on the effects of plant-based diet on virus causing diseases.

**References**	**Type of study**	**Type of vege diet adopted by recruited participant**	**Type of virus**	**Type of associated disease**	**Number and details of participants**	**Groupings (only control and vege group are listed)**	**Parameters measured**	**Outcome details**	**Conclusion on the effects of plant-based diet against virus causing diseases**	**Remarks**
Yedjou et al. (6)	Ecoloigcal study	Participant who adopted a high or low intake of vegetables or fruits (VF) determined from the recommendations according to the dietary guidelines	SARS-CoV-2	COVID-19	33 countries based on Kaggle website	10 developed countries - lowest VF consumption 7 developed countries - highest VF consumtption 17 developing countries - highest VF consumption	COVID-19 confirmed cases, deaths, recovered cases, and active cases	**In developed countries:** Low intake of VF had higher incidences and deaths rate Enough or more intake of VF had lower death rate even when the incidence rate was high **In developing countries:** Enough or more intake of VF had lower incidence and death rates	Positive	Diet data of countries, obtained from Kaggle website (https://www.kaggle.com/mariaren/covid19-healthy-diet-dataset) COVID-19 confirmed cases, deaths, recovered cases, and active cases obtained from Johns Hopkins Center for Systems Science and Engineering (CSSE) website.
Kim et al. (2)	Case-control study	Participant who adopted a whole foods, plant-based diet, vegetarian diet and/or pescatarian diet No definition otherwise	SARS-CoV-2	COVID-19	**Total: 2,884** **Gender** Male: 2,066 Female: 794 **Age** Average: 48 yrs	**Control:** Plant-based diets: 213 Plant-based diets or pescatarian diets: 248 **Case:** Plant-based diets: 41 Plant-based diets or pescatarian diets: 46	PCR or COVID-19 antibody test with or without COVID-19 symptoms	**OR COVID-19** (p < 0.05) Plant-based to not plant-based diet: 0.27 Plant-based or pescatarian diets to following either these 2 diets: 0.41 Low carbohydrate, high protein diet to plant-based diets: 3.55–3.96	Positive	COVID-19 patients following plant-based diets or a plant-based diets or pescatarian diet was associated with statistically significant lower odds of moderate-to-severe COVID-19-like illness compared with individuals who did not follow these diets.
Abdullah and Hassan (11)	Ecoloigcal study	Not defined	SARS-CoV-2	COVID-19	158 countries/states	Consumption of foods categorized into dietary factors including: fruits, non-starchy vegetables, beans and legumes, unprocessed red meats, and fruit juices	National dietary data from the Global Dietary Database of the United Nations and coronavirus disease statistics from the World Health Organization (WHO)	Mortality rate decreased with increased intake of non-starchy vegetables (p < 0.05) Infection rate decreased with high intake of beans and legumes (p < 0.05) Crude mortality rate decreased with increased consumtion of fruit (p < 0.05) and beans and legumes (p < 0.05) Crude mortality rate increased with increased consumtion of fruit juices (p < 0.05) and unprocessed red meats (p < 0.05) Crude infection rate increased with increased consumption of fruit (p < 0.001), fruit juices (p < 0.001), and unprocessed red meats (p < 0.001)	Positive andnegative	The present study showed higher intake of fruits and sugar-sweetened beverages (fruite juice) had a positive effect on infection and mortally rates by COVID-19, respectively. In contrast, the higher intake of beans and legumes had a negative effect on both increasing infection and mortality rates.
Chang et al. (12)	Case-control study	Participant who adopted a vegetarian habit of equal or more than 1 meal/week without food from animal sources for more than 1 year	HCV	Primary Liver Cancer (PLC) and HCC	**Total: 190** **Gender** All male **Age** 30–85 yrs	Vegetable consumption (control) <6 meals per week: 25 Vegetable consumption ≥6 meals per week: 162	anti-HCV antibodies	**Relative risk of HCV (univariate analysis)** Vegetable consumption (meals/week) of less than 6 to more (or equal) than 6: 3.26 (p < 0.05) **Relative risk of HCC (multivariate analysis)** Vegetable consumption (meals/week) of less than 6 to more (or equal) than 6: 17.77 (p < 0.05)	Positive	Anti-HCV seropositivity, HBsAg carrier status, vegetable consumption frequency, and history of chronic liver disease were included in the multivariate analysis as they are significantly associated with PLC
Tomita et al. (13)	Case-control study	Participant who adopted a dietary patterns of dark- green and deep-yellow fruits and vegetables.	HPV	cervical intraepithelial neoplasia (CIN)	**Total: 792** **Gender** All female **Age** 21–30 yrs: 275 31–40 yrs: 316 41–50 yrs: 291 51–60 yrs: 176	HPV negative (control): 331 HPV positive: 461	HPV genotyping and PCR detection of collected exfoliated cells (endocervical and endocervical region) Serum micronutrient level	**OR HPV positivity** (p < 0.05) High intake to low intake of vege diet: 0.51	Positive	Dietary intakes of dark green vegetables as well as fruits and their juices were significantly inverse associated with HPV positivity Strong inverse associations between serum lycopene and tocopherols, vege diet, as well as fruits and their juices with CIN and invasive cancer risks
Srivastava et al. (14)	Cross-sectional study	Not defined	HPV	Not mentioned	**Total: 2,424** **Gender** All female **Age** ≤ 25 yrs: 742 26–35 yrs: 1,298 36–45 yrs: 249 ≥46 yrs: 109	Non-vegetarian (control): 1,229 Vegetarian: 1,158	HPV genotyping and PCR detection of collected exfoliated cells (endocervical region)	**OR HPV positivity** (p < 0.05) Non-vegetarian to vegetarian: 1.31	Positive	Non-vegetarian diet showed a significant higher association with HPV (P < 0.05)

In a case–control study conducted by Kim et al. (2) on high-risk healthcare workers from six countries (France, Germany, Italy, Spain, UK, USA), the association between vegetable intake and severity of COVID-19 was studied (Table 1). A total of 568 high-risk healthcare workers infected with COVID-19 and 2,316 controls had 11 choices of diets to select from, including plant-based and pescatarian diets. The severity of COVID-19 among patients was determined based on the severity of symptoms. Their findings demonstrated that participants following plant-based diets and a combined category of plant-based and pescatarian diets had significantly lower odds of moderate to severe COVID-19 than those who did not follow such diets. These findings, however, may not reflect the outcomes of more severe COVID-19 cases as such patients were excluded from the study owing to their physical inability to participate in the questionnaire. Nevertheless, Kim et al.'s study highlights the possibility that regardless of the dietary type or dietary restrictions of an individual, an overall higher consumption of vegetables and a lower intake of poultry and red or processed meats possibly contribute to a reduced risk of severe COVID-19.

Despite the positive studies describing the benefits of high VF consumption in combating COVID-19 infections, a study by Abdullah and Hassan (11) demonstrated some contradicting results (Table 1). In their ecological study, the dietary data and COVID-19 disease statistics from 158 countries were obtained from the World Health Organization. First, the authors found that countries with a high intake of plant proteins, including beans and legumes, were significantly associated with a lower COVID-19 infection rate. This was in line with findings described in studies elsewhere (2, 15). However, they found that countries that consumed high amounts of fruit and fruit juices had a statistically significant increase in COVID-19 infection rates. The authors hypothesized that this unexpected outcome may be due to the high glycemic index of fruits, which may suppress the role of the immune system in combating pathogenic infections. They further described that the consumption of higher quantities of fruit indirectly suppresses the stimulation of antimicrobial proteins, defensins and cathelicidins, involved in the inhibition of virus replication, raising the levels of anti-inflammatory cytokines, and lowering the levels of pro-inflammatory cytokines. This suggests that although fruits are rich in micronutrients and bioactive compounds that promote the modulation of immune system functions, a high intake of fruits with a high glycemic index may exhibit negative effects in combating COVID-19 infections. These findings are still inconclusive as that study did not use a multivariate adjusted model to account for numerous confounding factors, such as age, BMI, or physical activity, that are likely factors that contribute to COVID-19 infection.

Besides respiratory viruses, cancer-causing viruses have attracted the attention of researchers to investigate how plant-based diets affect the outcome of the infection and progression of diseases.

## HCV

Primary liver cancer (PLC) is ranked the sixth most common cancer and the third most common cause of cancer mortality worldwide, with an estimated 906,000 new cases and 830,000 deaths in 2020 (16). Hepatocellular carcinoma (HCC) is the most common type of liver cancer and accounts for approximately 75% of all PLC (17). HCC is a primary malignancy, arising from hepatocytes, and at least 60% of HCC cases are attributed to hepatitis viruses (18). In recent decades, epidemiological evidence has shown that dietary factors can play a preventive role in some cancer types, including HCC (19).

An early study conducted on the male population in Taiwan sought to identify the association between increased vegetable consumption and HCV infection in PLC development (12). A total of 38 patients with PLC (characterized by the presence of anti-HCV in blood serum) and 152 healthy controls were interviewed for their vegetable consumption frequency, characterized by the number of meals with fresh vegetables taken per week, and whether they followed a vegetarian diet (Table 1). Their findings demonstrated a significant association between vegetable consumption and HCV infection, where participants with a weekly vegetable consumption of more than six meals had a significantly lower HCV infection risk than those who did not. Interestingly, they also observed a significant negative relationship between a vegetarian diet and HCC development, which may imply that a diet that reduces the intake of animal products may be beneficial in countering PLC development.

## HPV

Cervical cancer is the fourth most common malignancy among women worldwide, accounting for approximately 7% of all cancer cases in women (20, 21). Persistent infection with HPV, particularly of those high-risk genotypes, is considered the major cause of cervical cancer. The HPV is a virus that can be sexually transmitted, and high-risk HPV genotypes are found to be present in 99.7% of cervical cancer specimens. Nevertheless, studies show that having a diet high in FV can reduce the risk of cervical cancer in people with HPV.

Tomita et al. (13) conducted a case–control study among 792 women (461 cases and 331 controls) to investigate associations between serum micronutrient levels and dietary patterns with the risk of cervical intraepithelial neoplasia (CIN) and invasive cancer (Table 1). Dietary information was obtained through a food frequency questionnaire, and exfoliated cervical cells were collected to test for HPV variants. Fasting blood samples were also collected for serum micronutrient level analyses. Their study found a significant inverse association between the dietary intake of dark green and deep yellow VF with HPV positivity and CIN. Such a study outcome indicates that a plant-based diet can increase the prevention against HPV infection and reduce the risk of CIN. Consuming darker colored VF may provide more beneficial effects against viral infection and diseases.

In Srivastava et al. (14) performed a cross-sectional study involving 2,424 Indian women with asymptomatic HPV infection (Table 1). The prevalence of HPV infection was then compared between 1,229 nonvegetarians and 1,158 vegetarians. Their dietary information was obtained through a questionnaire, and cervical scrape samples were collected for HPV testing. Statistical analyses of the data showed a significant difference between HPV infection among nonvegetarians and vegetarians. The study therefore concluded a strong association between women with HPV infection on a nonvegetarian diet and those on a vegetarian diet.

## Discussion

Although numerous papers support the notion that plant-based diets can reduce the risk of viral infections and chronic diseases, there are several shared limitations of these studies. One apparent limitation in these studies is the non-uniform definition of vegetarianism. Throughout different studies, “vegetarianism” (and derivatives of the word) took on several meanings or was left undefined by the authors. For instance, although the study by Srivastava et al. (14) showed a significant association between vegetarianism and the risk of HPV infection, the terminology was never defined clearly in the paper. As highlighted by Dagnelie and Mariotti (22), “vegetarianism” is an umbrella term for all categories of diets that omit animal-based products. Variations of such diet include ovo-lacto-vegetarianism, pesco-vegetarianism, flexitarianism, and veganism, each of which may include different animal products in their diets, thereby possibly influencing the results of a study. Therefore, when it comes to the terminology, authors should clearly indicate the type of vegetarianism involved in their studies and adhere strictly to a specific category while performing said studies to eliminate confounding factors.

The correct sample size chosen is another important aspect in every study. A small sample size recruited may result in insufficient statistical power to draw statistically significant conclusions (23). Even though all the published studies reviewed in this article showed a promising potential of plant-based diets against disease-causing viruses, some studies showed a vast disparity in participant numbers between nonvegetarian and vegetarian groups; thus, this may lead to a nonstatistical significance because of lack of statistical power. Therefore, future studies should ensure that the number of participants recruited is large, and the sample size per group is approximately similar to provide more accurate and unbiased findings, a smaller margin of error, and adequate power to detect statistical significance among the groups compared.

Although it has been established that following vegetarian diets offers numerous health benefits, little research is available on the effects of the duration of vegetarianism and its health benefits (24). A recent study conducted by Jakše et al. (25) sought to identify the short-, medium-, and long-term effects of a whole-food vegetarian diet. A majority of their findings revealed no statistically significant difference between short-term (<2 years), medium-term (<5 years), and long-term (<10 years) vegetarians for cardiovascular risk factors. However, in particular, women following the diet for 5–10 years showed significant benefits in terms of higher total and HDL-cholesterol and lower triglycerides and LDL-cholesterol levels. Although Jakše et al.'s study focused on cardiovascular risk factors, it could reflect the overall benefits of a vegetarian diet. Hence, the duration in which one follows a vegetarian diet could be a factor affecting the benefits obtained. This aspect is, however, absent in the studies reviewed in this paper (2, 14). It is thus suggested that future studies should consider including the duration of adopting a vegetarian diet in the dataset to further enhance the study.

## Conclusion

Based on all these reported studies, adopting a plant-based diet has been shown to be able to reduce the risk of various virus causing diseases. Although scientific efforts are made to acknowledge the beneficial effects of plant-based diets against viral infections, several limitations still need to be addressed, for example, the sample size used, study type, and self-reported questionnaires, as these may influence the results of a study. Moreover, even the effectiveness of plant-based diets may vary according to the types of viruses. Therefore, more research with robust study designs and approaches are needed to reach a more definitive conclusion.

## Author Contributions

CW, CL, and HM prepared the manuscript. JT and HC conceived the idea, reviewed the drafts, and provided important information for the completion. CT reviewed the draft and provided important information for the completion. All authors approved the final version of this manuscript for submission.

## Funding

This study was supported by Honours Funding 2021 from School of Science, Monash University Malaysia (5220101).

## Conflict of Interest

The authors declare that the research was conducted in the absence of any commercial or financial relationships that could be construed as a potential conflict of interest.

## Publisher's Note

All claims expressed in this article are solely those of the authors and do not necessarily represent those of their affiliated organizations, or those of the publisher, the editors and the reviewers. Any product that may be evaluated in this article, or claim that may be made by its manufacturer, is not guaranteed or endorsed by the publisher.
